# Trends in incidence and mortality of nasopharyngeal carcinoma over a 20–25 year period (1978/1983–2002) in Sihui and Cangwu counties in southern China

**DOI:** 10.1186/1471-2407-6-178

**Published:** 2006-07-06

**Authors:** Wei-Hua Jia, Qi-Hong Huang, Jian Liao, Weimin Ye, YY Shugart, Qing Liu, Li-Zhen Chen, Yan-Hua Li, Xiao Lin, Fa-Lin Wen, Hans-Olov Adami, Yi Zeng, Yi-Xin Zeng

**Affiliations:** 1 Departments of Experimental Research, Cancer Center, Sun Yat-sen University, GuangZhou, State Key Laboratory of Oncology in Southern China, China; 2 Sihui Cancer Registry, Guangdong, China; 3 Cangwu Cancer Registry, Guangxi, China; 4 Department of Epidemiology and Statistics, Karolinska Insititutet, Sweden; 5 Department of Epidemiology, Johns Hopkins University, Baltimore, USA; 6 Institute of Virology, Chinese National Center for Disease Control, Beijing, China

## Abstract

**Background:**

Nasopharyngeal carcinoma (NPC) is a rare malignancy in most parts of the world but is common in southern China. A recent report from the Hong Kong Cancer Registry, a high-risk area for NPC in southern China, showed that incidence rate decreased by 29% for males and by 30% for females from 1980–1999, while mortality rate decreased by 43% for males and 50% for females. Changing environmental risk factors and improvements in diagnosis and treatment were speculated to be the major factors contributing to the downward trend of the incidence and mortality rates of NPC. To investigate the secular trends in different Cantonese populations with different socio-economic backgrounds and lifestyles, we report the incidences and mortality rates from two population-based cancer registries in Sihui and Cangwu counties from 1978–2002.

**Methods:**

Incidence and mortality rates were aggregated by 5-year age groups and 5 calendar years. To adjust for the effect of difference in age composition for different periods, the total and age-specific rates of NPC incidence and mortality rate were adjusted by direct standardization according to the World Standard Population (1960). The Estimated Annual Percentage Change (EAPC) was used as an estimate of the trend.

**Results:**

The incidence rate of NPC has remained stable during the recent two decades in Sihui and in females in Cangwu, with a slight increase observed in males in Cangwu from 17.81 to 19.76 per 100,000. The incidence rate in Sihui is 1.4–2.0 times higher during the corresponding years than in Cangwu, even though the residents of both areas are of Cantonese ethnicity. A progressive decline in mortality rate was observed in females only in Sihui, with an average reduction of 6.3% (p = 0.016) per five-year period.

**Conclusion:**

To summarize, there is great potential to work in the area of NPC prevention and treatment in southern China to decrease NPC risk and improve survival risk rates in order to reduce M:I ratios. Future efforts on effective prevention, early detection and treatment strategies were also discussed in this paper. Furthermore, the data quality and completeness also need to be improved.

## Background

The epidemiology of nasopharyngeal carcinoma (NPC) shows a uniquely skewed geographic distribution. The disease is rare in most parts of the world, where incidence rates are generally below 1 per 100,000 person-years [1]. However, in southern China and southeastern Asia, the rates can be as high as 20 to 50 per 100,000 person-years [1,2]. The highest risk has been observed in Cantonese of the Guangdong province, thus giving NPC a special name – "Canton tumor". However, no international report has been published on its secular trends in high-risk areas, except for Hong Kong.

Sihui city, located along the Xijiang River in the middle east of Guangdong province, has the highest incidence of NPC in China. In the 1970s, a cancer registry was established in Sihui to report the incidence and mortality of the major cancers, including NPC. Cangwu county is upstream of the Xijiang river in Guangxi province and is on the border between Guangxi and Guangdong. Given the high incidence of NPC in Cangwu, a cancer registry system was also established there in the 1980s.

Cancer Incidence in Five Continents (CI5) is regularly published by the International Agency for Research on Cancer (IARC), providing comparable data on the incidence of cancer in different geographical locations and distinct sub-populations (particularly ethnic) within these locations. However, none of the cancer registries in southern China (except Hong Kong) has taken part in the CI5 project. Until the year 2000, only a few Chinese registries such as Shanghai, Tianjin, Qidong, and Hong Kong have been involved in the project.

Recently, a very informative epidemiological study from the Hong Kong Cancer Registry reported that the age-standardized incidence rate of NPC in Hong Kong steadily decreased between 1980 and 1999; the total decrease amounted to 29% for males and 30% for females over the 20-year period [3]. This encouraging reduction of NPC incidence in Hong Kong has been attributed to the changing lifestyle among local residents from traditional Chinese to Western diets, followed by rapid economic growth and development, which started in early 1960s. These data suggested that reduction of exposure to environmental risk factors can reduce the incidence of NPC. In fact, a decreasing trend was also observed in the Shanghai female population, with moderate risk during the 23 years from 1972–1974 to 1993–1994[4]. A recent report on Chinese-Americans living in Los Angeles County and the San Francisco Metropolitan area also suggested that the rates decreased by 37% in men but by just 1% in women from 1992 to 2002, with the overall decline limited primarily to type I tumors. Type I represents well to moderately differentiated squamous cell carcinomas with keratin production, but among Cantonese or Chinese living in Hong Kong, Taiwan and Macao type III tumors predominate (undifferentiated carcinoma or lymphoepitheliomas) [5]. In Singapore, NPC incidence rates also started to decrease from the mid-1990s [6]. A nationwide report for time trends in cancer mortality in China showed that the age-standardized mortality rate of NPC also declined from 1987 to 1999, especially in urban areas [7].

To investigate the secular trends in NPC in different Cantonese populations with different socio-economic backgrounds and lifestyles, we examined the incidence of and mortality from NPC from 1978 to 2002 in Sihui and Cangwu counties to search for possible etiological clues and provide guidelines for primary and secondary prevention in a southern Chinese population.

## Methods

### Cancer registry

**Figure 1:**
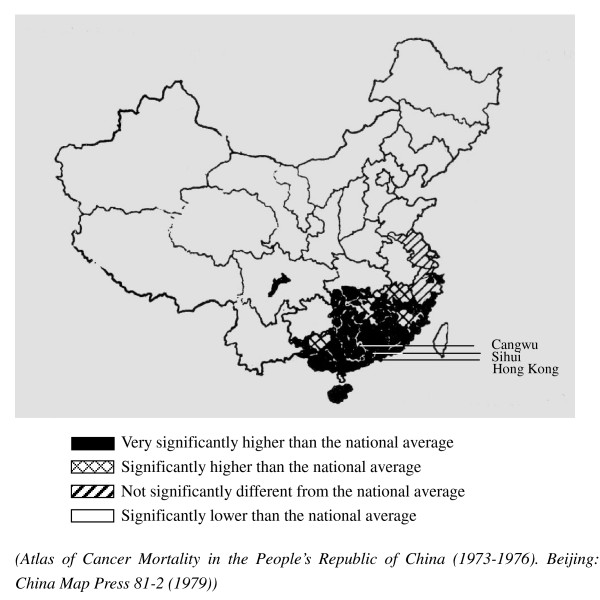
Map of China with NPC mortality indicated. The map shows the NPC mortality based on a nationwide investigation carried out from 1973–1976. Black areas indicate the regions with the highest mortality from NPC in southern China. Cangwu, Sihui, and Hong Kong are marked.

Sihui and Cangwu are two counties located in the Guangdong and the Guangxi province, respectively, in southern China. Both counties are located along the Xijiang river that originates from the mountainous region of the Guangxi province and eventually joins the Pearl River and the Southern China Sea (Figure 1, Map of China with Sihui, Cangwu, and Hong Kong indicated). In the year 2000, there were 393,655 and 649,404 inhabitants in Sihui and Cangwu, respectively. The populations in both counties are of Cantonese origin.

The Sihui and Cangwu Cancer Registries were established in 1977 and 1982, respectively. Since there was no law for compulsory reporting of cancer cases, and people could choose hospitals freely, a Cancer Prevention Network was formed by local governments. The basic unit is the village, in which local general practitioners are responsible for health care of the local residents. They reported incident cancer cases regularly (at least annually) to health officers in the regional hospital of each town. In general, each town consists of 10 to 20 villages, and each county consists of 10 to 20 towns or administrative districts. The cancer registry collected reports from each regional hospital or local clinical oncology departments of the county hospitals.

For each incident cancer case, information including registered identification number (ID), medical ID, China Identity Card Number (unique for each resident), ICD code (9^th ^or 10^th ^version), name, sex, birth date, occupation, ethnicity, resident address, phone number, cancer site, diagnosis basis, and pathological report if available (date of diagnosis, hospital and doctor name for diagnosis) are all registered. To ensure completeness of registration, the list of NPC patients enrolled in surrounding referral hospitals was also collected regularly. Table 1 shows the proportion of diagnoses with histological confirmation. Up to 85.2–90.3% and 95.5–97.3% of NPC patients were diagnosed by pathology in the Sihui and Cangwu Registry, respectively. The surviving NPC patients were followed up by a Cancer Registry staff member through regular interviews of the patients or their family members once a year by phone call, letter, or home visit until death occurred. They assigned the cause of death by "death certification" which was provided by the local public security bureau and the hospitals where patients were treated and/or died. Death information was also re-confirmed according to annual whole-population and whole-death causes report from local health bureau. For those who died of traffic accident, suicide or other non-NPC related disease, such as heart attack, stroke, the direct causes of death were recorded.

### Statistical analysis

The first register for incident cases of NPC in Sihui and Cangwu began in 1977 and 1982, respectively, so we used data from 1978 and 1983 to avoid over-reporting in the first year. Incidence and mortality rates were aggregated by 5-year age groups and 5 calendar years. To adjust for the effect of difference in age composition for different periods, the total and age-specific rates of NPC incidence and mortality rate were adjusted by direct standardization according to the World Standard Population (1960)[8].

The Estimated Annual Percentage Change (EAPC) [9] was used as an estimate of the trend. Using calendar year as a regression variable, a regression line was fitted to the natural logarithm of the rates, i.e. y=mx+b, where y=ln(rate) and x=calendar year. EAPC was calculated using the equation EAPC = 100×(e^m^-1). Testing the hypothesis that the EAPC is equal to zero is equivalent to testing the hypothesis that the slope of the regression line is zero, using the t-distribution of m/SE_m_. The number of degrees of freedom equals the number of calendar years minus 2. The standard error of m, i.e. SE_m_, is obtained from the fit of the regression line. This calculation assumes that the rates increased/decreased at a constant rate over the entire period.

The study was approved by the human ethical committee of Cancer Center, Sun Yat-Sen University.

## Results

### Incidence

From 1978–2002, a total of 1,710 incident NPC cases in Sihui were registered, including 1,159 males and 551 females. From 1983–2002, a total of 1,297 cases in Cangwu were registered, including 973 males and 324 females. Table 2 represents the age-standardized incidence rates for different genders during different periods in the two areas. For example, from 1998–2002, the rates were 30.94 in males and 13.00 in females in Sihui and 19.76 in males 7.33 in females in Cangwu per 100,000.

**Figure 2:**
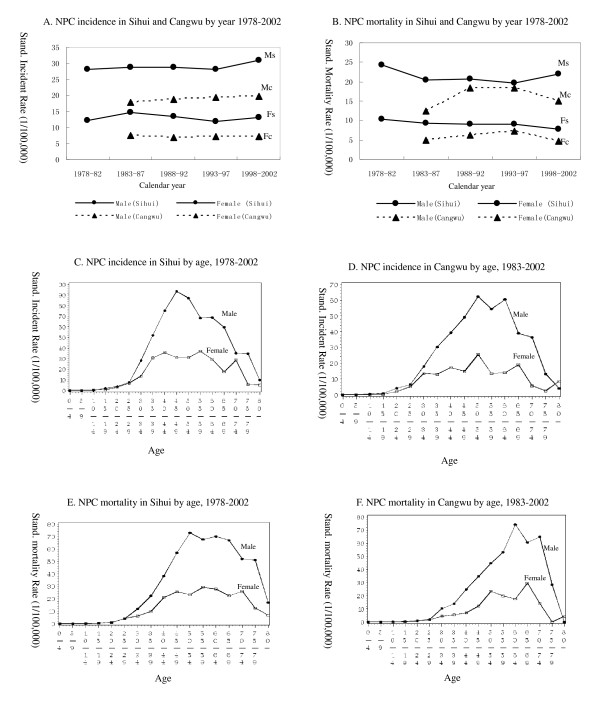
NPC incidence and mortality rates in Sihui (1978–2002) and Cangwu (1983–2002) by calendar year and age, standardized by world population. Ms (males in Sihui), Fs (Females in Sihui), Mc (Males in Cangwu), Fc (Females in Cangwu).

Table 2 and Figure 2A show that the incidence rates have remained stable in Sihui over 25 years (EAPC 1.73%, *P *= 0.215 *vs *.EAPC -1.12%, *P *= 0.739), whereas a slight increase was observed in males in Cangwu from 17.81 to 19.76 per 100,000 (EAPC 3.57%, P = 0.020), but the trend remain stable in females (EAPC 0.00%, *P *= 0.998) over 20 years. Moreover, the incidence in Sihui was 1.4–2.0 times higher during the same time period (Figure 2A).

NPC was very rare among populations younger than 30 years, but the rate then rose sharply to reach a peak at age 50–59 years in Cangwu. However, in Sihui the peak was advanced and prolonged, beginning at age 40–49 years and remaining constant until age 59 years (Table 2 and Figure 2C and 2D).

### Mortality

A total of 1,215 NPC cases died from 1978–2002 in Sihui, 827 males and 388 females, and 1,051 cases died from 1983–2002 in Cangwu, 778 males and 273 females. Table 2 summarizes the mortality rates for the different genders during different time periods in the two areas. We observed a progressive decline in females in Sihui from 10.47 to 7.73 with an average reduction of 6.32% (EAPC -6.32% *P *= 0.016) per five-year period, amounting to a total decrease of 26% (Table 2 and Figure 2B). This decline was also observed in males but did not reached significance (EAPC -2.56% *P *= 0.413). No consistent trends were observed in Cangwu. Age-standardized mortality rates are shown in Figures 2E and 2F.

## Discussion

Commonly suspected risk factors for NPC include Epstein-Barr virus (EBV) infection, environmental factors, and genetic susceptibility. EBV has been consistently identified as an important risk factor, with a dose-response relationship between EBV antibody level and NPC risk [10-13]. Consumption of Cantonese-style salted fish and preserved food, which contain high levels of nitrosamines, especially during weaning period and adolescence, has long been considered an important risk factor for NPC [14-20]. Cigarette smoking, occupational exposures to wood dust, formaldehyde, and chemical fumes, as well as use of Chinese herbs have also been associated with increased NPC risk [21,22]. Tea, especially green tea, has demonstrated to decrease the risk of several cancers. Green tea contains several components including catechins, a category of polyphenols that have chemopreventive properties. [23,24]. However, to date, no positive result has been report for the association of green tea with reduced NPC risk. In recent years, a series of studies have also provided evidence that the genetic component is a key etiological factor in the occurrence and development of NPC [25-29].

The most interesting findings from this study is that the incidence rates of NPC have largely remained stable in Sihui and Cangwu in the recent 20- to 25-year period (except for a slight increase in males of Cangwu), which differs from a consistent declining trend reported in Hong Kong. As we know, the risk of NPC varies not only in the world, but also in different sub-ethnicities within the Chinese. The IARC reports that the age-standardized incidence rate for the Chinese (adjusted by world population) was 4.46 for males in Shanghai (located in eastern China) and 1.61 in Tianjin (located in northern China) from 1988–92 [1]. Before, during, and after Hong Kong was returned to the mainland central government in 1997, many people from northern China moved to Hong Kong, and some people from Hong Kong, who are native Cantonese, moved to other countries. Whether or not the proportion of Cantonese in the total Chinese population has declined in recent years needs to be further investigated, because this could possibly contribute to the decreased rates. More advantage, Sihui and Cangwu have stable population structure, the report seems to reflect NPC secular trend in completely Cantonese.

The 1978 policy of "Reform and Open to Outside of the World" in China Mainland led to changes in both lifestyle and living conditions, which have influenced the cancer profile. For example, the incidence of lung cancer for males in Qidong, China increased from 32.3 to 48.5 per 100,000, while the incidence of stomach cancer decreased from 43.1 to 35.6 in 1983 and 1997, respectively [30]. In southern China, both Sihui and Cangwu are well known for their high incidences of NPC, and therefore preventive measures have been carried out there in the last two decades. The traditional diet has changed progressively, and some identified NPC risk factors in foods such as preserved salted fish are no longer frequently consumed in most households. Previous studies have also suggested that exposure to risk factors such as salted fish in infancy confers a higher NPC risk than in adulthood [22]. Accordingly, those who born after 1978 in the two areas were less than thirty years old during the period of this study and have not reached peak ages for incidence of NPC until now, the significant decline in NPC incidence unable to be observed. Moreover, in Singapore the NPC rates started to show a declined trend 30 years after economical development. The real economic development in China occurred in early 1990s and thus we anticipate the decreasing rate of NPC in China would occur in the next 10 to 20 years, given continued social and economic development and transitions in lifestyle. Surely, there are some possibilities related to registries themselves that could have an influence on the rates. For example, an improved cancer registry system that collecting more cancer cases that could be missed before, and improved diagnostic procedure on NPC that could be misdiagnosed before, etc.

An argument against this could be the fact that environmental risk factors should act as an accumulating effect, and 20 years is long enough to reflect some changes in incidence. Certainly, future studies are required to draw a clearer conclusion.

In our study, the incidence of NPC in Sihui was 1.4 to 2.0 times higher in males and females as compared to their counterparts in Cangwu. Cangwu is about 210 kilometer far from Sihui. Historically, it was part of the Guangdong province-Inhabitant in both areas is Cantonese sharing a similar genetic background and speaks the same dialect. Noteworthy, NPC incidences are quite different between the two areas. The reasons why NPC risk is different in two populations have not been thoroughly investigated and few comparative epidemiological study have been carried out until recent years. During recent decades, Sihui still has higher economic development speed than Cangwu, the latter is defined as a county of poverty in China, where inhabitant annual average income just as half as those in Sihui. Social economic level may reflect the variations in related to life style, diet habit, living condition as well as other factors. We also observed a decrease in mortality with an average reduction of 2.56% and 6.32% for males and females, respectively, per five-year period from 1978–2002 in Sihui, and although it did not reach a significant level in males, we are encouraged by the result. A screening program has been conducted in a high-risk population in Guangdong (including Sihui), and since 1986, 98,180 residents have participated in the program [31]. The study showed that the 5-year survival rate for NPC patients in this screening project was 79.87%, significantly higher than the 58.43% in hospital-based cases during the same period. We would like to note that the Sihui population has improved the importance and awareness of early diagnosis of NPC, -compared with those living in other areas, as a result of the implemented screening program.

In this paper, we analyzed the mortality-incidence ratios (M:I ratio) in two registries (Table 1). The M:I ratio, which compares the number of deaths attributed to a specific cancer and the number of incident cases in the same time period, can be interpreted as an indirect indicator of general survival if registration is complete and no marked temporal changes in incidence rates are present [32]. The ratios in Sihui have been stable since 1983 but fluctuated in Cangwu. We noted that mortality rates increased during the middle ten years (1988–1997) in Cangwu, which caused an increase in the M:I ratios. After further observation, we found that 51% of the new cases diagnosed from 1991–1995 died of the malignancy within one year after their diagnosis, while the 1-year survival rate was reported to be as high as 98.2% for new cases who received regular treatment during the same period [33]. Radiotherapy is a key therapy for NPC clinical treatment. We noticed that the price of radiotherapy has doubled in all hospitals in Cangwu since 1991. Some NPC patients might have found the increased costs unaffordable and terminate radiotherapy. On the other hand, this observation may also be due to lack of the completeness of Cangwu cancer registry during that period. In addition, the clinical staging of NPC at initial diagnosis may shed some light on this discrepancy, but we cannot deepen analysis because the records are not completeness in the registries.

## Conclusion

NPC is an important health problem in southern China. Effective prevention and treatment strategies need to be further developed, which will greatly benefit residents in high-risk areas. Future efforts could focus on several aspects: changing unhealthy traditional diet habits, developing screening programs or annual physical examinations including EBV serology tests, nasopharyngeal examinations in high-risk populations, improving clinical treatment strategies, making efforts to minimize delayed diagnosis, increasing the accuracy in staging, and promoting treatment strategies and making radiotherapy available for all NPC-patients at a lower cost. Moreover, basic scientific research on genetics, environment and their interaction should be conducted to clarify NPC aetiology in-depth.

## Competing interests

The author(s) declare that they have no competing interests.

## Authors' contributions

WHJ, paper design, data analysis and paper writing

QHH, in charge of whole work of cancer registration in Sihui registry

JL, in charge of whole work of cancer registration in Cangwu registry

WY, data analysis and manuscript revising

YYS, data analysis, interpretation and manuscript revising

QL, data analysis

LZC, data verification and cleaning

YHL, participated in work of Sihui registry

XL, participated in work of Sihui registry

FLW, participated in work of Sihui registry

HOA, participated in the design of the study

YZ, supervised and participated in work of Cangwu registry

YXZ, in charge of whole work related to the paper, final approval of the version to be publishedAll authors read and approved the final manuscript.

## Pre-publication history

The pre-publication history for this paper can be accessed here:



## Figures and Tables

**Table 1:** Proportion (%) of diagnoses type for NPC incident cases, Mortality *vs*. incidence (M:I) ratios for males and females during different time periods in the Sihui and Cangwu Cancer Registries

Period	Diagnoses type in Sihui			Diagnosis type in Cangwu			M:I Ratio in Sihui		M:I Ratio in Cangwu	
				
	Histological verification	Clinical deduction	Death certificate	Histological verification	Clinical deduction	Death certificate	Male	Female	Male	Female
1978–82	89.1	10.1	0.80	-	-	-	0.87	0.85	-	-
1983–87	89.6	9.50	0.90	95.5	2.41	2.06	0.71	0.64	0.70	0.67
1988–92	87.7	10.0	2.30	96.0	1.65	2.31	0.72	0.67	0.98	0.91
1993–97	85.2	13.0	1.80	96.5	2.02	1.44	0.70	0.76	0.95	1.00
1998–2002	90.3	8.90	0.80	97.3	1.06	1.60	0.71	0.59	0.76	0.65

**Table 2:** Age-standardized incidence and mortality rates of NPC per 100,000 during different time periods and the Estimated Annual Percent Changes (EAPC) in Sihui and Cangwu

	Period					EAPC (p value)
		
	1978–82	1983–87	1988–92	1993–97	1998–2002	
**Incidence Rate **(number of cases)						
Sihui						
Male	28.07 (167)	28.68 (205)	28.65 (235)	28.03 (268)	30.94 (284)	+1.73% (0.215)
Female	12.29 (91)	14.79 (111)	13.35 (116)	11.81 (116)	13.00 (117)	-1.12% (0.739)
Cangwu						
Male		17.81 (209)	18.68 (225)	19.43 (259)	19.76 (280)	+3.57% (0.020)
Female		7.44 (82)	6.91 (78)	7.23 (68)	7.33 (96)	0.00 (0.998)
						
**Mortality Rate **(number of cases)						
Sihui						
Male	24.42 (143)	20.49 (141)	20.65 (166)	19.56 (182)	21.95 (195)	-2.56% (0.413)
Female	10.47 (80)	9.43 (73)	8.95 (76)	9.01 (89)	7.73 (70)	-6.32% (0.016)
Cangwu						
Male		12.53 (137)	18.38 (213)	18.41 (227)	15.00 (201)	+5.56% (0.622)
Female		4.97 (54)	6.27 (71)	7.26 (87)	4.73 (61)	-0.019% (0.999)
